# Serum Mac-2 Binding Protein Glycosylation Isomer to Predict the Severity of Hepatic Fibrosis in Patients with Hepatitis C Virus Infection

**DOI:** 10.3390/diagnostics12112650

**Published:** 2022-10-31

**Authors:** Chen-Hua Liu, Chun-Jen Liu, Tung-Hung Su, Shang-Chin Huang, Tai-Chung Tseng, Jo-Hsuan Wu, Pei-Jer Chen, Jia-Horng Kao

**Affiliations:** 1 Department of Internal Medicine, National Taiwan University Hospital, Taipei 100225, Taiwan; 2 Hepatitis Research Center, National Taiwan University Hospital, Taipei 100225, Taiwan; 3 Department of Internal Medicine, National Taiwan University Hospital, Yun-Lin Branch, Douliou 640203, Taiwan; 4 Graduate Institute of Clinical Medicine, National Taiwan University College of Medicine, Taipei 100233, Taiwan; 5 Department of Internal Medicine, National Taiwan University Hospital Bei-Hu Branch, Taipei 108206, Taiwan; 6 Department of Medical Research, National Taiwan University Hospital, Taipei 100225, Taiwan; 7 Hamilton Glaucoma Center, Shiley Eye Institute and Viterbi Family Department of Ophthalmology, University of California, San Diego, CA 92039, USA

**Keywords:** hepatitis C virus, hepatic fibrosis, Mac-2 binding protein glycosylation isomer, liver stiffness, transient elastography

## Abstract

Large-scale studies to assess the utility of the Mac-2 binding protein glycosylation isomer (M2BPGi) in predicting hepatic fibrosis in patients with hepatitis C virus (HCV) infection are limited. Serum M2BPGi level determination was performed in 1460 patients with HCV who received liver stiffness measurement (LSM) using transient elastography (TE). The correlation of LSM and grade of hepatic fibrosis as staged by TE with M2BPGi was assessed. Receiver operating characteristic (ROC) curves were constructed to evaluate the diagnostic power of M2BPGi for fibrosis stages of ≥F2, ≥F3, and F4. The selected M2BPGi cutoff values were chosen based on the maximal Youden index, a positive likelihood ratio (LR) ≥ 10, and a negative LR ≤ 0.1. Serum M2BPGi level was highly correlated with LSM (Pearson correlation coefficient: 0.567, *p* < 0.001) and hepatic fibrosis stage (Spearman’s rank correlation coefficient: 0.772, *p* < 0.001). The areas under ROC curves (AUROCs) of M2BPGi for ≥F2, ≥F3, and F4 were 0.865 (95% confidence interval [CI]: 0.846–0.884), 0.937 (95 % CI: 0.922–0.952), and 0.962 (95% CI: 0.951–0.972). The maximal Youden indices for ≥F2, ≥F3, and F4 were 1.72, 2.65, and 3.93. By selecting M2BPGi cutoff values with a positive LR ≥ 10 and a negative LR ≤ 0.1, clinicians were able to correctly discriminate F2, F3, and F4 in 69.1%, 77.8%, and 90.1% of patients. In conclusion, serum M2BPGi is a good diagnostic tool to predict the severity of hepatic fibrosis in patients with HCV infection.

## 1. Introduction

Chronic hepatitis C virus (HCV) infection remains a significant health problem that affects approximately 0.7% of the world’s population [1]. Following chronic HCV infection, persistent hepatic inflammation can induce progressive hepatic fibrosis, which may result in cirrhosis, hepatocellular carcinoma (HCC), and hepatic decompensation [2,3,4]. In contrast, hepatic fibrosis can be halted or even regressed in most patients whose HCV is successfully eradicated with antiviral treatment [5].

The advent of direct-acting antivirals (DAAs) has revolutionized the care of HCV because nearly all patients can achieve viral eradication through a finite and short course of highly tolerable and effective therapy, particularly in the era of pan-genotypic DAAs [6,7,8,9]. Despite this tremendous progress in anti-HCV treatment, an accurate assessment of hepatic fibrosis is still needed to help clinicians optimize their therapeutic and surveillance strategies in daily practice [10,11,12].

To date, liver biopsy is the gold standard tool to stage the severity of hepatic fibrosis in patients with chronic liver diseases. However, the risks of postprocedural pain, infection, bleeding, and accidental injury of nearby organs preclude the widespread use of liver biopsy [13,14]. Moreover, sampling and interpretation variability of biopsy specimens frequently occur, which has led to the development of noninvasive means as surrogate makers for assessing hepatic fibrosis [15,16]. Various noninvasive tools, including biochemical, serological, and radiological indices, have been formulated to predict the severity of hepatic fibrosis in patients with HCV infection [17]. Although transient elastography (TE), a noninvasive tool to measure liver stiffness, is increasingly appealing to healthcare providers with its excellent diagnostic power to predict different stages of hepatic fibrosis for HCV, it is expensive and not readily available in general medical facilities [18].

Mac-2 binding protein (M2BP) is a 92 kilodalton cell-adhesive protein of the extracellular matrix (ECM), which possesses a feature of self-oligomerization to form a large “sweet-doughnut-like” ring structure covered with N-acetylgalactosamine (GalNAc), and is thus named M2BP glycosylation isomer (M2BPGi) [19,20,21]. Mac-2 (galectin-3) is secreted by monocytes and macrophages in response to HCV-induced hepatic injury and can initiate trans-differentiation of quiescent hepatic stellate cells (HSCs) to an active phenotype. In addition to laying down ECM to promote hepatic fibrosis, these activated HSCs also secret M2BPGi, which serves as the juxtacrine-acting messenger to Kupffer cells during hepatic fibrogenesis [20]. Because there is a strong link between serum M2BPGi and hepatic fibrogenesis, studies have shown that serum M2BPGi levels are associated with fibrosis stage, dynamic fibrosis evolution, antiviral responses, presence of esophagogastric varices, risk of HCC occurrence or recurrence, extrahepatic malignancy, and survival in patients with HCV infection [19,22,23,24,25,26,27,28,29,30,31,32,33,34,35,36].

Although serum M2BPGi levels are associated with the severity of hepatic fibrosis in patients with HCV infection, the diagnostic accuracy as well as the cutoff levels of M2BPGi selected to predict different stages of hepatic fibrosis vary significantly across studies, which may be attributed to sample size, hepatic fibrosis distribution, and the adoption of reference standards [19,22,23,24,25,32]. To this end, we conducted a large-scale study to confirm and validate the clinical utility of serum M2BPGi in diagnosing hepatic fibrosis in patients with HCV infection.

## 2. Materials and Methods

### 2.1. Patients

We conducted a retrospective study, enrolling patients with chronic HCV infection who received liver stiffness measurement (LSM) with transient elastography (FibroScan^®^, Echosens, Paris, France) at the National Taiwan University Hospital (NTUH) and NTUH Yun-Lin Branch before antiviral treatment and for whom serum samples had been stored between January 2012 and December 2021 and could be used to determine the serum M2BPGi level. All serum samples were stored at −80 °C until analysis. Chronic HCV infection was defined as the presence of detectable HCV antibody (anti-HCV; Abbott HCV EIA 2.0, Abbott Laboratories, Abbott Park, IL, USA) and quantifiable serum HCV RNA (Cobas TaqMan HCV Test v2.0, Roche Diagnostics GmbH, Mannheim, Germany, lower limit of quantification [LLOQ]: 15 IU/mL) for ≥6 months. Patients who had hepatitis B virus (HBV) or human immunodeficiency virus (HIV) coinfection; chronic kidney disease stage 5, which was defined as an estimated glomerular filtration rate (eGFR) <15 mL/min/1.73 m^2^; decompensated cirrhosis (Child-Pugh B or C); a history of HCC; organ transplantation; or failed or unreliable LSM by TE, or who refused or were unable to provide written informed consent, were excluded from the study.

### 2.2. Study Design

We collected patients’ baseline demographics, including age, sex, history of diabetes mellitus (DM), arterial hypertension (HTN), dyslipidemia, body mass index (BMI), and waist circumference. Blood tests including hemogram, international normalized ratio (INR), serum albumin, total bilirubin, alanine aminotransferase (ALT), creatinine, fasting glucose, insulin, glycosylated hemoglobin (HbA1c), triglyceride, total cholesterol, high-density lipoprotein (HDL), low-density lipoprotein (LDL), anti-HCV, HBV surface antigen (Abbott Architect HBsAg qualitative assay, Abbott Laboratories, Abbott Park, Illinois, USA), anti-HIV (Abbott Architect HIV Ag/Ab Combo, Abbott Laboratories, Abbott Park, Illinois, USA), HCV RNA, and HCV genotype (Abbott RealTime HCV Genotype II, Abbott Laboratories, Abbott Park, IL, USA) were also assessed [37]. The upper limit of normal (ULN) of ALT level was 30 U/L for men and 19 U/L for women [38]. The eGFR and the CKD stage were calculated and graded via chronic kidney disease-epidemiology collaboration (CKD-EPI) equation [39,40]. Metabolic-dysfunction-associated fatty liver disease (MAFLD) was defined by the international expert consensus statement, including three different phenotypes (overweight or obesity; lean/normal weight; type 2 DM) [41]. Patients with LSM values of ≤7.0 kPa, 7.1–9.4 kPa, 9.5–12.4 kPa, and ≥12.5 had fibrosis stages of F0–F1, F2, F3, and F4, respectively [42,43].

Serum M2BPGi levels were quantified using a Wisteria floribunda agglutinin (WFA)-antibody sandwich immunoassay with an automated HISCL-800 immunoanalyzer (Sysmex Co., Kobe, Japan). The level was expressed as cutoff index (COI) using the following equation: ([M2BPGi]_sample_ − [M2BPGi]_NC_)/([M2BPGi]_PC_ − [M2BPGi]_NC_), where NC and PC denote negative and positive controls.

### 2.3. Statistical Analysis

The statistical analyses were performed using the Statistical Program for Social Sciences (SPSS Statistics Version 23.0, IBM Corp., Armonk, NY, USA). When appropriate, the baseline characteristics are shown as median (interquartile range, IQR) and number (percentage). We analyzed the relationship between serum M2BPGi level and LSM with Pearson correlation, and that between serum M2BPGi level and hepatic fibrosis stage (F0–F1, F2, F3, and F4) with Spearman’s rank correlation [18,43]. Receiver operating characteristic (ROC) curves were constructed for M2BPGi. The areas under ROC curves (AUROCs) with 95% confidence interval (CI) of M2BPGi are shown according to fibrosis stages of significant hepatic fibrosis (≥F2), advanced hepatic fibrosis (≥F3), and cirrhosis (F4) [44]. We also assessed the AUROCs of M2BPGi in subgroups of interest, including age > 60 years, male sex, MAFLD, HCVRNA > 2,000,000 IU/mL, HCV genotype 1, ALT > 2 folds ULN, and CKD stage 3 or 4. Three selective cutoff values of M2BPGi to predict fibrosis stages of ≥F2, ≥F3, and F4 were chosen: (1) the maximal Youden index with a maximal value of (sensitivity + specificity − 1); (2) the index with a negative likelihood ratio (LR) ≤ 0.1; (3) index with a positive LR ≥ 10. For each selective cutoff value, we showed the sensitivity, specificity, positive predictive value (PPV), negative predictive value (NPV), positive LR, negative LR, and accuracy. All statistics were two-tailed and results were considered statistically significant when the *p* value was <0.05.

## 3. Results

### 3.1. Patient Characteristics

**Figure 1:**
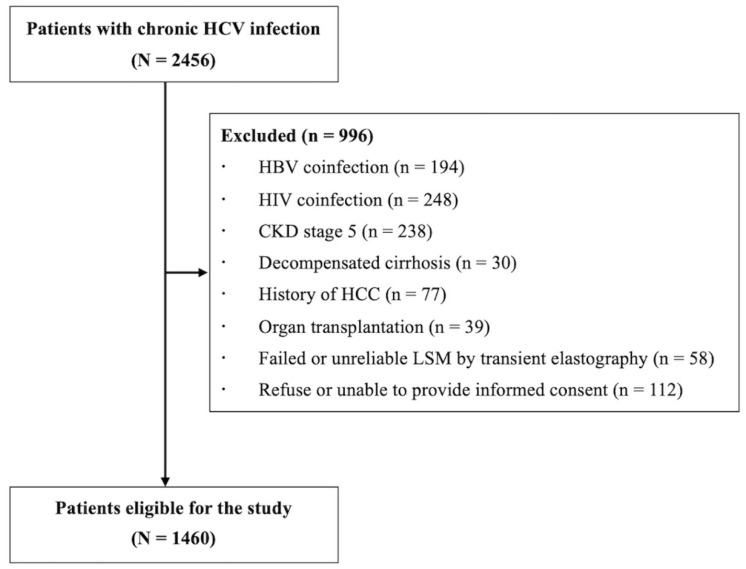
Study workflow.

Of 2456 patients with chronic HCV infection, 1460 patients were eligible for the study after 996 were excluded because of HBV coinfection (*n* = 194), HIV coinfection (*n* = 248), CKD stage 5 (*n* = 238), decompensated cirrhosis (*n* = 30), a history of HCC (*n* = 77), organ transplantation (*n* = 39), failed or unreliable LSM (n = 58), or refusal or inability to provide written informed consent (*n* = 112) (Figure 1).

Table 1 shows the baseline patient characteristics. In total, 447 (30.6%), 795 (54.5%), 748 (51.2%), 1074 (73.6%), and 1149 (78.7%) patients were aged > 60 years, males, and had MAFLD, BMI ≥ 23 kg/m^2^, and ALT > 2-fold ULN, respectively. The median levels of M2BPGi and LSM were 1.81 COI and 7.6 kPa. By LSM, 559 (38.3%), 410 (28.1%), 179 (12.3%), and 312 (21.4%) patients had a fibrosis stage of F0–F1, F2, F3, and F4. Five hundred and three (34.5%), 863 (59.1%) patients, and 251 (17.2%) had a baseline HCV RNA > 2,000,000 IU/mL, HCV genotype 1 infection, and CKD stage 3 or 4.

### 3.2. Correlation between Serum M2BPGi Level and Hepatic Fibrosis

**Figure 2:**
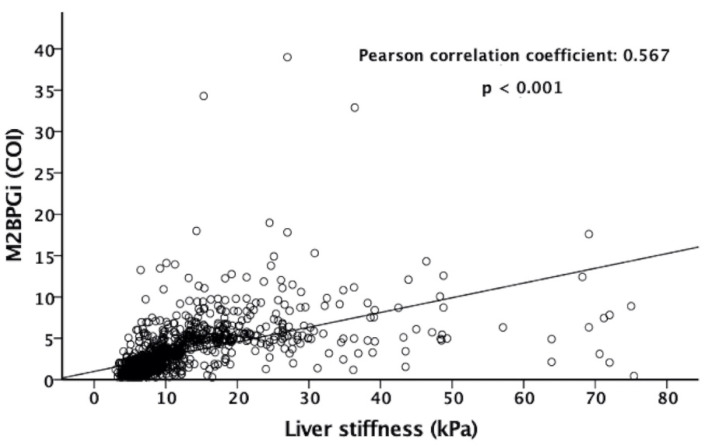
Scatter plot of serum M2BPGi level (COI) and LSM (kPa) with TE. The Pearson correlation efficient was 0.567 (*p* < 0.001).

**Figure 3:**
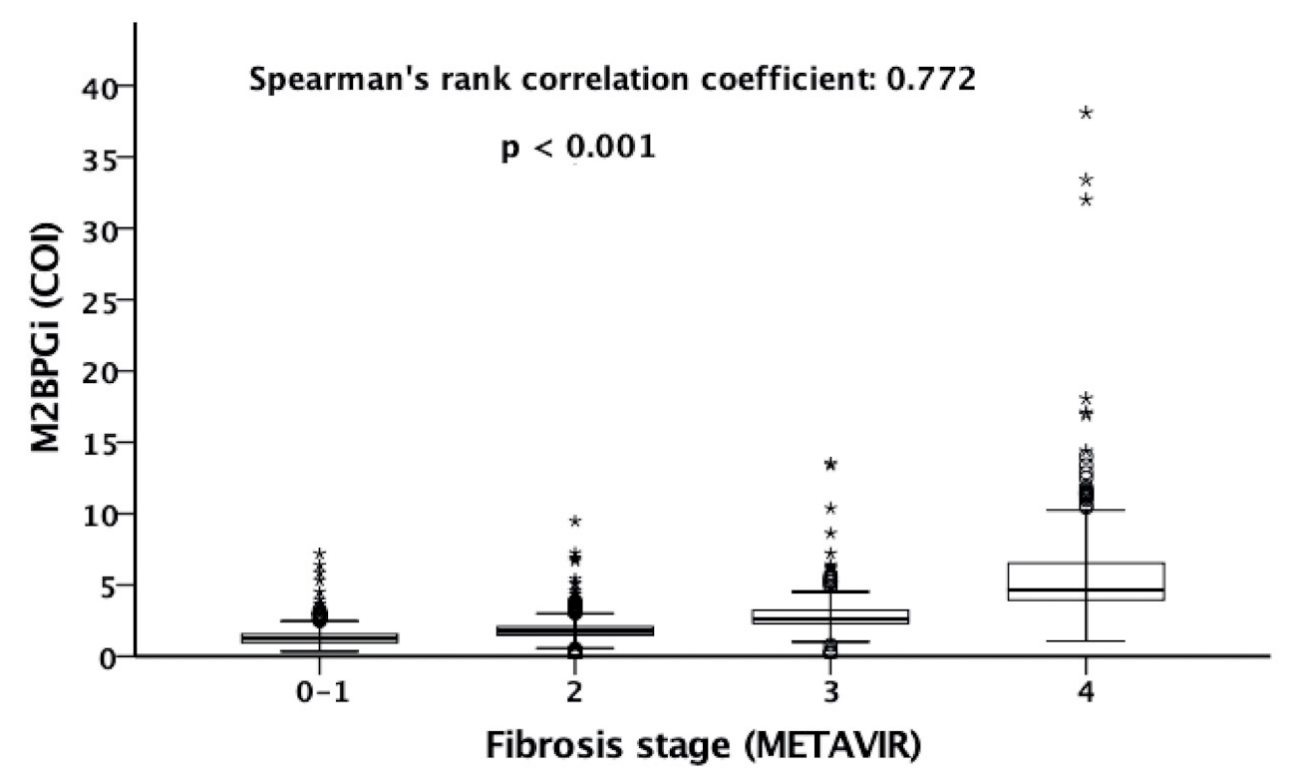
Box plots of serum M2BPGi level (COI) for METAVIR hepatic fibrosis F0–1, F2, F3, and F4 as categorized by LSM. The tops and bottoms of the boxes are the first and the third quartiles. The tops and bottoms of the horizontal lines are the upper and lower whiskers. The circles denote mild outliers and the asterisks denote extreme outliers. The Spearman’s rank correlation coefficient was 0.772 (*p* < 0.001).

The serum M2BPGi level was significantly correlated with LSM (Pearson correlation coefficient: 0.567, *p* < 0.001) (Figure 2). Figure 3 shows the box plots of serum M2BPGi level according to METAVIR fibrosis stages of F0–F1, F2, F3, and F4 as determined by TE. The median (IQR) values of M2BPGi levels for F0–F1, F2, F3, and F4 were 1.06 (0.77–1.38), 1.72 (1.45–2.01), 2.18 (1.87–2.82), and 4.82 (4.09–6.54), respectively (Figure 3). The Spearman’s rank correlation coefficient between M2BPGi and the grade of hepatic fibrosis was 0.772 (*p* < 0.001).

### 3.3. AUROC of M2BPGi to Predict the Severity of Hepatic Fibrosis

**Figure 4:**
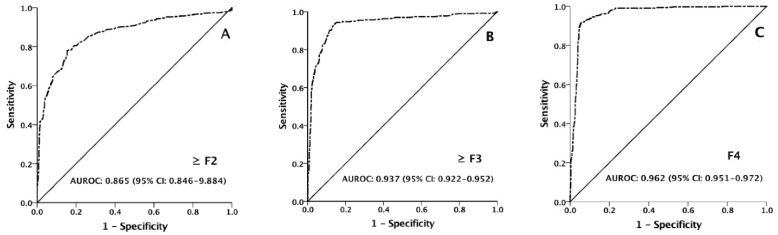
ROC curves of M2BPGi in predicting patients with fibrosis stages of ≥F2, ≥F3, and F4. The AUROCs were 0.865 (95% CI: 0.846–0.884) for ≥F2 (**A**), 0.937 (95% CI: 0.922–0.952) for ≥F3 (**B**), and 0.962 (95% CI: 0.951–0.972) for F4 (**C**), respectively.

The AUROCs of M2BPGi were 0.865 (95% CI: 0.846–0.884), 0.937 (95% CI: 0.922–0.952), and 0.962 (95% CI: 0.951–0.972) in predicting patients with fibrosis stages of ≥F2, ≥F3, and F4 (Table 2, Figure 4A–C). Among patients in subgroups of interest, the AUROCs ranged from 0.860–0.892 in predicting a fibrosis stage of ≥F2, 0.918–0.949 in predicting a fibrosis stage of ≥F3, and 0.935–0.971 in predicting a fibrosis stage of F4 (Table 2).

### 3.4. Selective M2BPGi Cutoff Values to Predict the Severity of Hepatic Fibrosis

The maximal Youden indices of M2BPGi in predicting patients with fibrosis stages of ≥F2, ≥F3, and F4 were 1.72, 2.65, and 3.93, with sensitivities of 81.3%, 88.1%, and 98.1%; specificities of 80.6%, 85.1%, and 95.0%; PPVs of 87.1%, 75.0%, and 84.3%; NPVs of 72.9%, 93.4%, and 99.5%; and accuracies of 81.1%, 86.2%, and 95.7%, respectively (Table 3). For diagnosing ≥F2, the M2BPGi cutoff index of 1.51 had a negative LR of 0.10, sensitivity of 92.4%, and a NPV of 95.9%; the cutoff index of 2.08 had a positive LR of 10.0, specificity of 93.5%, and a PPV of 94.2%. For diagnosing ≥F3, the M2BPGi cutoff index of 2.48 had a negative LR of 0.10, sensitivity of 92.2%, and a NPV of 95.1%; the cutoff index of 2.87 had a positive LR of 10.1, specificity of 91.6%, and a PPV of 83.1%. For diagnosing F4, the M2BPGi cutoff index of 3.50 had a positive LR of 10.04, sensitivity of 98.7%, and a NPV of 99.6%; the cutoff index of 4.35 had a negative LR of 0.1, specificity of 95.6%, and a PPV of 85.4% (Table 3). When we combined the selected M2BPGi cutoff values with a negative LR ≤0.10 and a positive LR of ≥10, 69.1%, 77.8%, and 90.1% patients could be correctly diagnosed for F2, F3, and F4.

## 4. Discussion

In line with the published reports, we confirmed that serum M2BPGi level was highly correlated with LSM and hepatic fibrosis stage as determined with TE in our large-scale study [19,20,22,23,24,25]. However, the AUROCs of serum M2BPGi level in predicting patients with fibrosis stages of ≥F2, ≥F3, and F4 varied significantly among various studies. The AUROCs in our study for predicting fibrosis stages of ≥F2, ≥F3, and F4 were 0.865, 0.937, and 0.962, and were superior to the reported AUROCs of 0.6–0.79 for ≥F2, 0.83–0.84 for ≥F3, and 0.76–0.89 for F4 in several studies [19,22,23,24]. However, the AUROCs in our study were similar to those in a report from Thailand which showed AUROCs of 0.86, 0.93, and 0.96 in predicting fibrosis stages of ≥F2, ≥F3, and F4 [25]. While the AUROCs of M2BPGi in predicting fibrosis stages of ≥F2 and ≥F3 in Kuno et al.’s study were lower than ours, the AUROC in predicting F4 was 0.96, which was identical to our report [19]. In other studies, the divergent AUROCs may be explained by relatively small sample sizes, recruitment of heterogeneous populations, and different cutoff levels of LSM used to define the stage of hepatic fibrosis [19,22,23,24,25].

We further examined whether some specific patient characteristics, including old age, male sex, coexistence of MAFLD, high HCV load, HCV genotype, elevated serum ALT level, or more advanced CKD stage, might significantly affect the AUROCs. The diagnostic performance of serum M2BPGi in these subgroups remained similar to the overall population, implying that the accuracy of M2BPGi was not compromised in patients with these factors. Based on the excellent diagnostic performance, serum M2BPGi may serve as a good noninvasive mean to assess hepatic fibrosis in patients with HCV infection.

Regarding the selection of optimized cutoff values for serum M2BPGi, we performed a comprehensive analysis by choosing the maximal Youden index with the highest level of sensitivity plus specificity, the cutoff point with a positive likelihood ratio ≥10.0 which provided strong evidence to rule in disease, and the cutoff point with a negative likelihood ratio ≤0.10 which provided strong evidence to rule out disease [45,46]. The diagnostic accuracy of serum M2BPGi levels with the maximal Youden indices tended to increase from 81.1% for ≥F2, 86.2% for ≥F3, to 95.7% for F4, and was in line with other noninvasive indices where the discrimination power increased with increasing severity of hepatic fibrosis [47,48,49,50]. In daily practice, it would be more informative and helpful for clinicians to exclude or include a defined stage of hepatic fibrosis. Our report demonstrated that M2BPGi cutoff values of less than 1.51, 2.48, and 3.50 can exclude the presence of ≥F2, ≥F3, and F4 with NPVs of 95.9%, 95.1%, and 99.6%. However, M2BPGi cutoff values of more than 2.08, 2.87, and 4.35 can suggest the presence of ≥F2, ≥F3, and F4 with PPVs of 94.2%, 83.1%, and 85.3%. Yamasaki et al. indicated that the mean serum levels of M2BPGi were 1.3, 2.2, 3.3, and 5.2 with fibrosis stages of F0–F1, F2, F3, and F4 in 541 patients receiving liver biopsy, which supported our recommendation to set the cutoff values with high PPVs and NPVs [32]. Interestingly, in concert with various reports showing an increased risk of HCC occurrence or recurrence in patients with serum M2BPGi level >1.70–2.00, we set a cutoff value of 2.08 to suggest the presence of ≥F2, which met the guideline recommendations to set a fibrosis stage of F2 for HCC surveillance [10,11,12,28,30,33,34]. Yamasaki et al. showed that the risk of HCC progressively increased when the serum M2BPGi level was stratified as <1.00, 1.00–4.00, and ≥4.00, and corresponded to our observations of F0–1, F2–3, and F4 with the optimized M2BPGi cutoff values [32]. Moreover, Kikukawa et al. proposed the presence of esophagogastric varices in patients with HCV if the serum M2BPGi level was >7.3, implying that cirrhotic patients with a cutoff value of >4.35 in our study may present significant portal hypertension due to more severe hepatic fibrosis [27].

The strengths of our study include (1) a sizable number of patients used for the analysis; (2) a homogeneous population achieved by excluding potential factors that might affect serum M2BPGi levels, such as HCC, organ transplantation, decompensated cirrhosis. HBV or HIV infection, and CKD stage 5; (3) application of TE, which has been demonstrated to be a good reference standard for assessing hepatic fibrosis. However, our study has several limitations. First, this was a retrospective study, which used archived serum samples to determine the serum levels of M2BPGi. Therefore, it remains elusive whether the diagnostic accuracy of serum M2BPGi would be affected using archived samples. Second, we cannot extrapolate our results to patients excluded from the analysis. Third, we did not use liver biopsy, which is seldom performed now with the rapid development and the widespread use of noninvasive means, as the reference standard. Lastly, we cannot discriminate in our study a minority of patients concomitantly presenting with drug-induced liver injury (DILI), autoimmune liver disease, hereditary hemochromatosis, or Wilson’s disease, which might affect liver fibrosis.

In conclusion, our large-scale study indicated that serum M2BPGi level was highly correlated with LSM and stage of hepatic fibrosis. The diagnostic performance of serum M2BPGi increased with the increasing severity of hepatic fibrosis. Using the optimized cutoff values with high positive LR and low negative LR for serum M2BPGi, 69.1%, 77.8%, and 90.1% patients with HCV infection could be discriminated according to fibrosis stages of F2, F3, and F4 using this simple serological index.

## Figures and Tables

**Table 1:** Baseline patient characteristics.

Characteristics ^a^	Patient (N = 1460)
**Age, years**	56 (47–62)
**Age > 60 years, *n* (%)**	447 (30.6)
**Male, *n* (%)**	795 (54.5)
**DM, *n* (%)**	329 (22.5)
**MAFLD, *n* (%)**	748 (51.2)
**HCV RNA, log_10_, IU/mL**	6.00 (5.31–6.51)
**HCV RNA > 2,000,000 IU/mL, *n* (%)**	503 (34.5)
**HCV genotype 1, (%)**	863 (59.1)
**LSM, kPa ^b^**	7.6 (6.3–11.3)
**Fibrosis stage (METAVIR), *n* (%) ^c^**	
F0–F1	559 (38.3)
F2	410 (28.1)
F3	179 (12.3)
F4	312 (21.4)
**M2BPGi, COI**	1.81 (1.26–3.24)
**BMI, kg/m^2^**	25.2 (22.8–27.7)
**BMI ≥ 23 kg/m^2^, *n* (%)**	1074 (73.6)
**Platelet count, 10^9^/L**	170 (130–210)
**INR**	1.00 (0.95–1.05)
**Albumin, g/dL**	4.2 (4.1–4.4)
**Total bilirubin, mg/dL**	0.9 (0.7–1.1)
**ALT, ULN ^d^**	3.79 (2.26–6.27)
**ALT > 2-fold ULN, *n* (%)**	1149 (78.7)
**eGFR, mg/dL/1.73 m^2 e^**	74 (64–86)
**CKD stage, *n* (%) ^f^**	
1	312 (21.4)
2	897 (61.4)
3	238 (16.3)
4	13 (0.9)

MAFLD, metabolic-dysfunction-associated fatty liver disease; HCV, hepatitis C virus; RNA, ribonucleic acid; LSM, liver stiffness measurement; kPa, kilopascal; M2BPGi, Mac-2 binding protein glycosylation isomer; COI, cutoff index; BMI, body mass index; INR, international normalized ratio; ALT, alanine transaminase; eGFR, estimated glomerular filtration rate; CKD, chronic kidney disease. ^a^ Data are shown as median (interquartile range, IQR) unless otherwise indicated. ^b^ Assessed via transient elastography. ^c^ The cutoff values of LSM for a hepatic fibrosis stage of F0–1, F2, F3, and F4 are ≤7.0 kPa, 7.1–9.4 kPa, 9.5–12.4 kPa, and ≥12.5 kPa, respectively. ^d^ The upper limit of normal (ULN) was 30 U/L for men, and 19 U/L for women. ^e^ Calculated via Chronic Kidney Disease Epidemiology Collaboration (CKD-EPI) equation. ^f^ According to eGFR cutoff values with CKD-EPI equation.

**Table 2:** The areas under the receiver operating characteristic (AUROCs) of M2BPGi in predicting the severity of hepatic fibrosis.

Overall Population/Subgroup	Fibrosis Stage					
	≥F2		≥F3		F4	
	AUROC	95% CI	AUROC	95% CI	AUROC	95% CI
**Overall (N = 1460)**	0.865	0.846–0.884	0.937	0.922–0.952	0.962	0.951–0.972
Age > 60 years (*n* = 447)	0.884	0.846–0.921	0.918	0.890–0.946	0.935	0.913–0.958
Male (*n* = 795)	0.860	0.834–0.886	0.935	0.913–0.957	0.971	0.959–0.982
MAFLD (*n* = 748)	0.870	0.844–0.896	0.944	0.925–0.963	0.963	0.950–0.976
HCV RNA > 2,000,000 IU/mL (*n* = 503)	0.892	0.863–0.920	0.927	0.898–0.956	0.970	0.951–0.989
HCV genotype 1 (*n* = 863)	0.883	0.861–0.906	0.948	0.931–0.965	0.959	0.944–0.973
ALT > 2 folds ULN (*n* = 1149)	0.870	0.849–0.891	0.937	0.922–0.953	0.955	0.943–0.968
CKD stage 3 and 4 (*n* = 251)	0.865	0.818–0.911	0.949	0.922–0.977	0.971	0.951–0.990

AUROC, area under the receiver operating characteristic; M2BPGi, Mac-2 binding protein glycosylation isomer; CI, confidence interval; MAFLD, metabolic-dysfunction-associated fatty liver disease; HCV, hepatitis C virus; RNA, ribonucleic acid; ALT, alanine transaminase; CKD, chronic kidney disease.

**Table 3:** Selective cutoff values of Mac-2 binding protein glycosylation isomer (M2BPGi) for prediction of the severity of hepatic fibrosis.

**Significant Hepatic Fibrosis (≥F2)**										
**M2BPGi (COI) ^a^**	**Patient Tested, *n* (%)**	**Actual Fibrosis, *n* (%)**		**Sensitivity** **(%)**	**Specificity** **(%)**	**PPV** **(%)**	**NPV** **(%)**	**Positive** **LR**	**Negative** **LR**	**Accuracy** **(%)**
	**All (N = 1460)**	**≥F2 (*n* = 901)**	**<F2 (*n* = 559)**							
1.72 (maximal Youden index)	841 (57.6)	733 (81.3)	108 (19.6)	81.3	80.6	87.1	72.9	4.19	0.24	81.1
1.51	1018 (69.7)	833 (92.4)	135 (24.2)	92.4	75.8	81.8	95.9	3.82	0.10	86.1
2.08	621 (42.5)	585 (64.9)	36 (6.5)	64.9	93.5	94.2	62.4	10.0	0.38	75.9
**Advanced hepatic fibrosis (≥F3)**										
**M2BPGi (COI) ^a^**	**Patient Tested, *n* (%)**	**Actual Fibrosis, *n* (%)**		**Sensitivity** **(%)**	**Specificity** **(%)**	**PPV** **(%)**	**NPV** **(%)**	**Positive** **LR**	**Negative** **LR**	**Accuracy** **(%)**
	**All (N = 1460)**	**≥F3 (*n* = 491)**	**<F3 (*n* = 969)**							
2.65 (maximal Youden index)	577 (39.5)	433 (88.1)	144 (14.9)	88.1	85.1	75.0	93.4	5.90	0.14	86.2
2.48	685 (46.9)	453 (92.2)	232 (23.9)	92.2	76.1	66.1	95.1	3.92	0.10	81.5
2.87	480 (32.9)	399 (81.2)	81 (8.4)	81.2	91.6	83.1	90.6	10.1	0.20	88.2
**Cirrhosis (F4)**										
**M2BPGi (COI) ^a^**	**Patient Tested, *n* (%)**	**Actual Fibrosis, *n* (%)**		**Sensitivity** **(%)**	**Specificity** **(%)**	**PPV** **(%)**	**NPV** **(%)**	**Positive** **LR**	**Negative** **LR**	**Accuracy** **(%)**
	**All (N = 1460)**	**F4 (*n* = 312)**	**<F4 (*n* = 1148)**							
3.93 (maximal Youden index)	363 (24.9)	306 (98.1)	57 (5.0)	98.1	95.0	84.3	99.5	19.62	0.02	95.7
3.50	421 (28.2)	308 (98.7)	113 (9.8)	98.7	90.2	73.2	99.6	10.04	0.01	92.0
4.35	329 (22.5)	281 (90.1)	48 (4.2)	90.1	95.6	85.4	97.3	20.48	0.10	94.6

M2BPGi, Mac-2 binding protein glycosylation isomer; COI, cutoff index; PPV, positive predictive value; NPV, negative predictive value; LR, likelihood ratio. ^a^ Youden index is defined as the value of sensitivity + specificity-1. The other two selective cutoff values of M2BPGi were chosen based on the positive LR ratio of ≥10 and the negative LR of ≤0.1, respectively.

## Data Availability

Data for this study, though not available in a public repository, can be made available upon reasonable request.
